# Spine Biomechanics in the Work of Aristotle (384 – 322 BC)

**DOI:** 10.1177/15533506221148012

**Published:** 2022-12-26

**Authors:** Christos Tsagkaris, Konstantinos Kalachanis, Jonas Widmer, Mazda Farshad

**Affiliations:** 1 Department of Orthopaedics, 31031 Balgrist University Hospital, Switzerland; 2 Spine Biomechanics, Department of Orthopaedics, 31031 Balgrist University Hospital, Switzerland; 3 Academic Affairs Department, 121340 New York College, Greece

**Keywords:** spine biomechanics, aristotle, philosophy, humanities

## Abstract

**Background:** Spine biomechanics is a field of applied research aiming to unravel the biomechanical understanding of the spine and its disorders and to understand the implications of their interventional therapy to improve clinical practice, physical performance and daily living. Its scientific whereabouts can be traced in the work of Aristotle, who discussed physical and biological concepts of spine biomechanics in a series of treatises.

**Results: **The authors searched the Thesaurus Linguae Graecae archive for original texts written in Greek and attributed to Aristotle and selected excerpts of medical and biological treatises that elaborate on spine biomechanics.

**Discussion: **While many of his theories have become outdated, his methodology and rationale remain relevant for contemporary researchers and clinicians. Here, the relevant content of passages of the corpus aristotelicum related to spine biomechanics and discuss their practical implications are presented.

## Introduction

The term “biomechanics” derives from the ancient Greek words βιο- (bio < bios=life) and μηχανική (mechanics). It is the application of engineering principles to living organisms, from humans, animals and plants to the functional units of life, the cells.^1^ Spine biomechanics use mechanical principles to study the vertebral column and its anatomically and functionally adjacent structures. During the last decades, it has evolved into a multidisciplinary research field with multiple applications in the management of spine conditions and even in ambient assistive living, ergonomics and sports science.^2^ Evidence of early understanding and investigation in spine biomechanics can be traced back to the Edwin Smith papyrus (2600-2200 B.C.) containing a number of case reports interpreted by means of spine biomechanics. Similar clinical – oriented evidence has been found in multiple medicinal texts of Persian, Indian, Greek, Roman and Arab origin attributed to eminent physicians such as Hippocrates, Galen and Avicenna, less known or unknown authors.^2^ Aristotle was probably 1 of the first to approach spine biomechanics through the lenses of physics and biology. Not only this is yet to be acknowledged in scientific literature, but most importantly his approach provides relevant reflection for contemporary research and clinical practice. Here, relevant content of passages of the corpus aristotelicum related to spine biomechanics are summarized and their current practical implications are discussed.

Aristotle, was born in 384 BC in Stagira, Macedonia region, Greece. His father served as a physician in the court of king Amyntas III of Macedonia. Although well versed in Medicine, Aristotle chose to study Philosophy in the renown Academy of Plato in Athens. After his studies he served as a tutor of Alexander the Great (356-323 BC), who later supported his biological research by providing him with animal and plants specimens from Asia and Africa. Aristotle’s work spans over major areas of human inquiry, including physics, biology, ethics and political science. His work has culminated in approximately 200 books including 9 books on anatomy and 2 books on medicine, 31 of whom have survived to date.^3-5^

## Methods

The authors searched the Thesaurus Linguae Graecae (TLG) archive for original texts written in Greek and attributed to Aristotle. The search focused on excerpts including possible references to the spine and its biomechanics. The selection of excerpts was made on the following criteria: 1) the relevance to the topic of spine biomechanics as perceived in the 21^st^ century; 2) the survival of the whole or the greatest part of the surrounding text, enabling the authors and other contemporary researchers to reach safe conclusions about the content they studied, and 3) the biological or medical orientation and focus of the excerpts– texts related to philosophy, theology and arts were not examined. Citations were obtained from credible and widely accessible modern editions, some of whom also provide translations of the original ancient Greek text into modern European languages.

## Results

**Figure 1.**
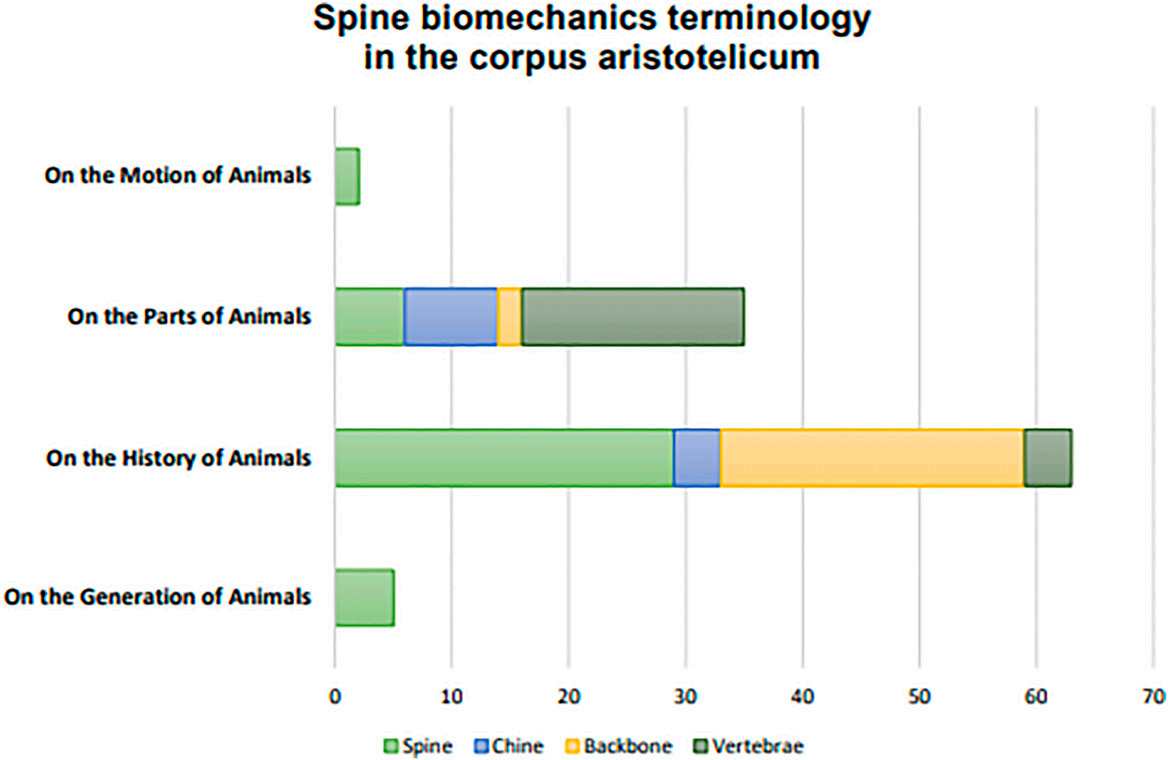
Quantitative presentation of spine biomechanics terminology in the corpus aristotelicum.

Aristotle’s perception of spine biomechanics is articulated in Generation of Animals (De generatione animalium),^6^ Movement of Animals (De motu Animalium),^7^ Parts of Animals (De Partibus Animalium),^8^ History of Animals (Historia animalium).^9^ The bulk of Aristotle’s knowledge and hypotheses are presented in the “Parts of Animals” and elaborated with examples across species in the “History of Animals”, while the “Generation” and “Movement of Animals” provide further context regarding the embryological, physical and ethical properties of motion in animals. A quantitive analysis of terms related to spine biomechanics in these books can be found in Figure 1.

In the “Motion of Animals”, Aristotle elaborates on the mechanical principles of movement and concludes that the spine is responsible for the symmetric movement of vertebrates. The spine is aligned with the middle line and acts as a geometric center, the geometric center of gravity in modern terms.^5^ In the “History of Animals”, the philosopher presents the spine as the point of origin of the osseous system, in view of its mechanical contribution to the movement of the head and the extremities.^6^ Despite its embryological inaccuracy, this assumption indicates that Aristotle held biomechanics in a high regard. In principle, Aristotle correlated the origin of an organ to its significance to the whole-body function – eg the heart would be the first organ to be formed because of its primordial role in blood circulation and in the same frame and the bones would originate from the spine because of its cardinal influence on musculoskeletal biomechanics.^6,7^ In the “Parts of Animals” Aristotle suggests that the spine is segmented in vertebrae, in order to increase the flexibility of the body. The segmentation not only enables various patterns of movement (flexion, stretch, bow, lordosis etc.), but it also protects the content of the spine (bone marrow, neural tract) from excessive mechanical loading and damage.^8^

Spine biomechanics serves also as a starting point for comparative anatomy; in vertebrates the spine maintains the shape of the body and facilitates movement, since Aristotle hypothesizes that even invertebrates should have a spine – like structure of different shape and composition to serve the same mechanical purpose.^8^ Going some steps further, Aristotle uses spine biomechanics to explain the adaptation of different animal species to the physical environment. According to him, larger animals have osseous, rigid and more dense vertebrae for weight bearing. Spine - like structures (made of less dense bone or cartilage) are deemed sustainable in smaller animals, increasing their flexibility, their ability to rotate their heads to detect and attack to predators (snakes) or to escape from them (eels). Nevertheless, environmental factors, namely high external pressure and cold temperatures make the spine of small animals more rigid. Aristotle justifies this assumption with the example of sea – urchins, whose spine is rigid as a countermeasure to the underwater pressure.^8^ The concept of spine biomechanics as a trait adapted to survival, is expanded in the “History of Animals” where crayfishes' and eels’ spines were compared on the basis of their predator – prey competition.^9^

Overall, Aristotle’s approach to spine biomechanics seeks the common principles that characterize the posture and movement of living organisms. Critical reasoning is employed in order to identify and interpret similarities and differences between the function of the spine or spine – like structures in different animals.

## Discussion

Aristotle emphasizes spine biomechanics on the grounds of biology and physics. Although Aristotle himself was the son of a physician and grew up studying medical textbooks, his approach differs significantly from medical texts of the period.^5^ Eminent physicians who lived before Aristotle, such as Hippocrates and his accolades focus on diseases of the human spine, and particularly on spine compression. In 2 instances, Hippocrates (ca 460 – 370 BC) mentions that the displacement of vertebrae can cause pain and voice alterations making the voice similar to the talk of dying man.^10-12^ The former describes pain related to spondylolisthesis, while the latter has probably been observed in lethal injuries involving the cervical spine. In both cases the descriptions consist of empiric observations, rather than pathogenetic or even philosophical explanations. Physicians who lived after Aristotle appeared to be more knowledgeable of the anatomy of the spine and commented in depth on the atlantoaxial joint and its support to the movement of the head. This is reasonable, because permissions for dissections on cadavers was granted for the first time by the Hellenistic monarchs of Alexandria, Egypt after the 3^rd^ century BC.^13^ The majority of relevant observations is based on the work of Galen (ca 130 – 200 AD), an eminent physician who treated Roman gladiators, had extensive experience with various type of injuries and was able to make anatomical observations during this work.^14,15^ Physicians in the eastern Roman empire, such as Oribasius of Pergamon (ca 325 – 403 AD), echoed the theories of Galen and built upon the observations of Hippocrates regarding voice alterations in individuals with spine injuries.^15,16^ Overall, it appears that, in contrast to Aristotle, physicians of the time concentrated on diseases of the spine and explored spinal anatomy to the extent possible and necessary for the improvement of clinical practice. Even their engagement with relevant philosophical work of Plato was strictly related to anatomy and did not delve into philosophical thinking.^17^

Certainly, the rather intellectual account of Aristotle includes a number of exaggerations or misconceptions with regard to the embryology and physiological purpose of the spine in the course of life. He also retrieves most of his examples from animals, which is interesting in terms of comparative anatomy but limits the deeper understanding of human spine biomechanics. Nevertheless, given that in the era of Aristotle cadaveric dissections were prohibited, it appears that he used as many animal specimens as possible in order to make his theory more comprehensive. As a matter of fact, his model of spine biomechanics can be applied to most species, including humans, considering their body composition and living environment (abductive reasoning). In this frame, Aristotle attempted to correlate spine biomechanics with the physical environment and essentially the quest for survival. Although he was not aware of the concept of evolution, he presented the formation and properties of the spine as a potential survival advantage. In the language of Aristotle, this corresponds to *endelecheia* and *telos*, the belief that its entity follows a trajectory that leads to the accomplishment of an inherent existential inclination towards survival and self – sustainability. In other words, he was able to trace fragments of evolutionary adaptation in spine biomechanics, but explained them by means of existential purpose (teleology).

Although several of Aristotle’s theories have become obsolete, contemporary researchers and clinicians can benefit from his focus on purpose, his abductive reasoning and his multidisciplinary approach.

• Keeping in mind that the purpose of spine biomechanics is to assist daily living and improve human performance and quality of life can serve as a guide for translational research (from the bench to the bedside approach) and empathetic clinical practice. Both are critical components of biopsychosocial care, that are at risk of being neglected in highly technical and technological advanced research, such as spine biomechanics.^18^
• Abductive reasoning can help address spine surgery failure. Despite the development of sophisticated instrumentation and techniques, a number of complications (adjacent segment degeneration, pseudarthrosis etc.) are still challenging patients and physicians. A growing body of evidence indicates that biomechanical risk factors and abnormalities contribute to such complications.^19^ Abductive reasoning can help researchers group observations of biomechanical abnormalities in imaging and posture in comprehensive pathogenetic models and hypotheses.^20^
• Spine biomechanics is already a multidisciplinary field prompting the cooperation of physicians, engineers and material scientists. Collaboration with behavioral and social scientists can help unravel the social and cultural underpinnings of posture and their implications on spine conditions. Collaboration with occupational health services can also help decrease the biomechanical risk factors leading to spinal deformities and promote an ergonomic lifestyle.

## Conclusion

Aristotle helped establish a scientific approach to the understanding of spinal biomechanics. Particularly, he highlighted the need to explore the mechanisms of posture and movement and their rational connection, in order to complement clinical experience and observations. While many of his theories have become outdated, his methodology and rationale remain relevant for contemporary researchers and clinicians.
